# Hiding in plain sight: the absence of consideration of the gendered dimensions in ‘source’ country perspectives on health worker migration

**DOI:** 10.1186/s12960-021-00571-6

**Published:** 2021-03-24

**Authors:** Ivy Lynn Bourgeault, Vivien Runnels, Jelena Atanackovic, Denise Spitzer, Margaret Walton-Roberts

**Affiliations:** 1 School of Sociology and Anthropology, University of Ottawa, Ottawa, ON K1N 6N5 Canada; 2 Gender, Work & Health Lab, University of Ottawa, Ottawa, ON K1N 6N5 Canada; 3 School of Public Health Sciences, University of Alberta, Edmonton, AB Canada; 4 Balsillie School of International Affairs, 67 Erb Street West, Waterloo, ON N2L 6C2 Canada

**Keywords:** Health worker migration, Gender dimensions, Source country perspectives, Policy responses

## Abstract

**Background:**

Gender roles and relations affect both the drivers and experiences of health worker migration, yet policy responses rarely consider these gender dimensions. This lack of explicit attention from source country perspectives can lead to inadequate policy responses.

**Methods:**

A Canadian-led research team partnered with co-investigators in the Philippines, South Africa, and India to examine the causes, consequences and policy responses to the international migration of health workers from these ‘source’ countries. Multiple-methods combined an initial documentary analysis, interviews and surveys with health workers and country-based stakeholders. We undertook an explicit gender-based analysis highlighting the gender-related influences and implications that emerged from the published literature and policy documents from the decade 2005 to 2015; in-depth interviews with 117 stakeholders; and surveys conducted with 3580 health workers.

**Results:**

The documentary analysis of health worker emigration from South Africa, India and the Philippines reveal that gender can mediate access to and participation in health worker training, employment, and ultimately migration. Our analysis of survey data from nurses, physicians and other health workers in South Africa, India and the Philippines and interviews with policy stakeholders, however, reveals a curious absence of how gender might mediate health worker migration. Stereotypical views were evident amongst stakeholders; for example, in South Africa female health workers were described as “preferred” for “innate” personal characteristics and cultural reasons, and in India men are directed away from nursing roles particularly because they are considered only for women. The finding that inadequate remuneration was as a key migration driver amongst survey respondents in India and the Philippines, where nurses predominated in our sample, was not necessarily linked to underlying gender-based pay inequity. The documentary data suggest that migration may improve social status of female nurses, but it may also expose them to deskilling, as a result of the intersecting racism and sexism experienced in destination countries. Regardless of these underlying influences in migration decision-making, gender is rarely considered either as an important contextual influence or analytic category in the policy responses.

**Conclusion:**

An explicit gender-based analysis of health worker emigration, which may help to emphasize important equity considerations, could offer useful insights for the health and social policy responses adopted by source countries.

## Introduction

Who migrating health workers are, why they migrate and the consequences of their migration are importantconsiderations in health workforce and migration policy design and implementation. Although there has been a visible growth in knowledge that gender roles and relations affect health workers’ reasons for migrating and their migration experiences [28, 65, 76, 77, 87, 96], there is little acknowledgement that gender is an important consideration in health worker migration policies. This lack of explicit attention from source country perspectives to these important gender dimensions can lead to inadequate and potentially inappropriate policy responses.

This paper builds on growing evidence of a *feminisation of migration* [16] where women constitute 44.3% of the estimated 244 million international migrants world-wide [52]. Although some countries have restricted women’s migration as a result of patriarchal ideologies and policy [41], increasingly women migrants are moving independently of partners or families [42, 81]. Migration is thus becoming better understood as highly gendered in that it impacts men’s and women’s roles and experiences of migration differently [75, 78, 87]. Although migration studies have generally become more gender analytic in nature [22, 76, 77], the literature remains modest on certain issues pertaining to gender and migration policy.

The feminization of migration is particularly striking for health workers who are themselves disproportionately female, and with female nurses encompassing the dominant health worker migrant group [13, 86]. Indeed, women migrants are over-represented globally in health and care sectors in which care-work has typically been undervalued because of its gendered female nature [27]. Nurses and other female health workers are disproportionately impacted by government austerity and cutbacks to health-care investment given their predominance in the sector [73]. Yet, while the migration of women as skilled health workers contributes significantly to global labour markets and migration patterns, as George notes, the “*gendered effects of migrant health workers on their personal and professional lives within the context of specific health occupations and specific recipient and source health systems*” [28], p. 37) remains poorly understood.

Undertaking an explicit gender-based analysis starting with binary sex disaggregation of data (female versus male)^1^ of migrant health workers can help in the interpretation of differences in women’s and men’s migration motivations and experiences. A more complex and nuanced view of gender does much more when it takes into consideration the social roles, relationships, behaviours, relative power and other traits that societies generally ascribe to women, men, and people of diverse gender identities [32, 72]. Further, migration decisions are not only influenced by gender at the individual and household level, but also at the institutional (training and employment) and policy level [9]. The gendered nature of the division of labour in health care [39, 44], for example, and how that is uniquely configured in both source or sending and recipient countries also has implications for understanding better the gendered nature of health worker migration and its impacts.

In the country-based empirical analyses we undertook of some of the key global health worker source or sending countries of the Philippines [17], India [90], and South Africa [46], we found many indications of how gender plays an important yet unexplored role in the causes, consequences and policy responses. In this paper, we explicitly draw out the gendered dimensions of health worker migration from the three key data sources—country-based documents, health worker surveys, and interviews with key stakeholders.

A brief note about terminology is important before we continue. In this paper, we use the term *migration* to refer to all forms of temporary and permanent movement across borders, or international mobility. This term encapsulates the migration out of countries—*emigration—*and migration into countries—*immigration,* that is, of source and destination country perspectives, respectively. Migration can also include internal movements, but this is not our focus here. Unless the topic is about explicit decisions to leave a country or from a source country perspective, where we use *emigration,* or when the topic is about entering and integrating into a specific country, where we use *immigration*, we will use *migration* where this is not specified.

## Gender and health worker migration

### Gendering push–pull factors influencing health workers decision to migrate

There is a tendency in the health worker migration literature to focus on individual level factors emphasizing how certain ‘push’ and ‘pull’ factors influence personal decisions to migrate by individuals and families [9]. Factors pushing health workers to migrate include poor wages, limited opportunities for professional development, heavy workloads, economic instability, poorly funded health-care systems, the burdens and risks of HIV/AIDS and safety concerns [45, 58, 68, 80, 93]. Pull factors include better and more comfortable living and working conditions, higher wages and greater opportunities for advancement and promotion [3, 14, 20, 93].

The push-pull approach to migration is intuitively appealing, but it provides little distinction of the relative importance of different factors, nor analytic attention to root causes at a more structural level, including the role of state and non-state intermediaries. This approach neglects the historical nature of colonial and post-colonial relations between countries which influence individual and collective decisions to migrate [31]. It also fails to fully account for the interactions of gender with factors in the broader political economy and the subsequent implications of those intersections at the household level, where decisions around migration and labour (paid and unpaid) are made [9, 41, 85].

Gender is intricately implicated in the migration decision of health workers through the gender-based discrimination and inequality experienced at home and abroad, which is perpetuated by traditional societal attitudes towards women and the care-work they undertake (e.g., [1, 15, 70]). Factors typically characterized as push, such as poor working conditions and low earnings particularly amongst occupations where women predominate, are often indicators of women migrants’ labour market marginality [65]. Moreover, the global demand for care-workers coupled with the expectation that women should earn for their families’ well-being—a discourse that is underscored by public declarations that migrant workers sustain the national family—help propel out-migration [76].

The Public Services International’s participatory research on migration and women health-care workers conducted in a number of countries demonstrated that women health workers are beset in a number of ways. Foremost, structural health sector reforms have a disproportionate negative effect on women health workers, who are subject to inequitably low wages and violence in the workplace, while at the same time they are often old enough to hold full responsibility for the care and support of their families [63, 64]. It is, as Susan Reverby [67] described, a caring dilemma—being forced to care in a context that does not value that care. All these factors can converge to compel women to migrate often leaving families behind, yet bearing the responsibility for their economic livelihood through remittances.

The gendered nature of transnational social networks, which increase female health workers’ awareness of immigration opportunities, also have a unique influence on their decisions [30, 47, 53, 70]. Ryan’s [70] work on Irish nurses who emigrated to Britain in the postwar period, for instance, reveals that most of them were encouraged to emigrate by female relatives, especially sisters, aunts and cousins. Adhikari [1] similarly illustrates how female nurses in Nepal are encouraged to migrate by women in their families: their emigration is seen as a collective family investment, the key return on investment being remittances. Scholars are increasingly recognizing how gender intersects with various drivers of the migration decisions of health workers.

### Gendered impacts of health worker emigration from source countries

The emigration of health workers can have positive impacts both formally on the health-care sector and informally on other sectors. For individual health workers, emigration can bring about gains in social and professional status, and these can be accentuated for women. Indian nurses who hold visas to work overseas, and therefore have the potential to emigrate, become preferred as potential wives because they can supply their own dowry, earn a wage, and buy a ticket for their future husbands [61, 84]. When confronted with situations of under- and unemployment, endemic in the female dominated health-care sector in sending countries, emigration offers significant opportunities for health workers to apply their training in an international context [39].^2^ Families ‘at home’ can also benefit from the significant contribution that remittances make to source country household incomes.

Reports of the negative impacts of migration, however, dominate the literature. Health worker shortages resulting from emigration can have adverse effects on care delivery, and in turn population health and health equity in source countries [17, 46, 82, 90]. The emigration of health-care workers can mean that source countries lose their investments in public education and training, which are further compounded with later loss of income tax revenue from highly skilled workers [31, 39]. How gender impacts these shortages is less clear: that nurses are the largest group of health professionals and that the majority of nurses are women means that migrating female nurses are lost to the sector, but filling the workload gaps that result from job vacancies and staff shortages, also falls to (predominantly) female nurses. While the levels of violence against women health workers have already been acknowledged as high in many countries (Cooper and Swanson nd) [34, 54], there is potential for additional harmful consequences. For example, some authors have suggested that as a result of their mobility away from their country of training, women workers face increased workload with attendant stress and low morale [39, 58].

Migration may improve social status of some female nurses [29], but it exposes others to different degrees of deskilling, in part as a result of intersecting racism and sexism in destination countries (e.g., [66]). Although likely to be migrating for their own economic purposes, female migrant health workers are vulnerable to certain experiences that male migrants do not experience, including increasing risks to their own health [40]. In addition to such vulnerabilities, according to the International Labour Organization (ILO) and Public Services International (PSI), there is a clear trend of undervaluing women’s work across professions [50].

Women’s decision to migrate, particularly if viewed as self-interested, is not without criticism. Female nurses can receive gender specific condemnation from others for abandoning or neglecting family in their pursuit of financial gain [36]. When female health workers emigrate, there are consequences for other women both within and outside of the health sector. Women’s gendered care roles are typically taken over by other women—older, younger or of lower status—who carry the burden of increased care responsibilities for sick or elderly family members in the context of weakened and under-resourced health systems [2, 24, 39, 51, 55]. Surrogate parenting for children is often provided by siblings and other female family members [24, 39]. The concept of global care chains, coined by Hochschild [35], and applied to the case of nurses by Yeates [94, 95] recognizes the role of female migrants as carers abroad, the care deficit left to be filled by women back home to whom their care responsibilities are transferred.

### Gendered nature of source country emigration policies

Studies of labour migration have often treated source countries as “unimportant auxiliaries” [60], merely reacting to the demands of the more powerful receiving nation-states which consume their citizens’ labour. This under-theorization of what the sending state does before the migrant leaves, and the impact of sending state policies on the skills composition, geographical reach and scale of international migration, remains an important research gap in the migration literature [48], and particularly as it relates to health workers.

Policy changes that have taken place in both source and destination countries are instrumental in precipitating or tempering women’s migration [6]. Although international human rights’ laws affirm that an individual has the right to leave and return to one’s own country [83] Article 13 (2), some countries have placed gender-based limitations on emigration based on law or social norms. As Ferrant et al. [26] explain, “High levels of discrimination against women reduce their ability to migrate … [and] deprive women of the resources necessary for cross-border migration.” (p. 4).

Examination of states’ emigration and immigration policies, cultural norms that foster international migration in some cases and prevent it in others, and the extent of women’s autonomy in making decisions to emigrate, provides additional evidence of the importance of examining gender influence explicitly. Restrictions or guidelines on women migrating are often specific to their gender, whereas restrictions placed on men are more likely to be targeted at their job class/profession. Emigration restrictions on women from Asian countries have included: banning of recruitment of certain sectors dominated by women, e.g. domestic helpers; restrictions on age of female migrants; selective bans on employment depending on destination country; and requirements of educational qualifications before exit permission is granted [57, 89].

Little of this literature focuses on the specific case of source country policy responses to the emigration of their domestically trained health workers, regardless of the utilization of an explicit gender lens. Even international guidance for both source and destination countries from the World Health Organization [92] in the form of its global code of practice on the international recruitment of health personnel fails to make visible sex and gender-based analyses [10, 12]. Invisible in particular are the perspectives in sending countries, experiences of health workers, and the lack of knowledge of gender and gender-based analysis by stakeholder decision-makers. These gaps clearly have implications for migration policy development and health workforce planning.

In summary, the literature of health worker migration has begun to show and acknowledge that gender is an important determinant of migration affecting the decision to emigrate, the experiences of migrants during the migration process, and the formal and informal impact on source and destination countries. The exploration of these issues remains relatively limited with respect to whether gender is evident in policy responses.

## Methods

The data that formed the basis of this paper are derived from the comparative research project “Source Country Perspectives on the Migration of Highly Trained Health Personnel”. Ethical approval and the methods that were employed for country-based studies have been reported in detail in the published literature to date [17, 46, 90], and thus are not replicated in this paper. The primary focus of the larger study was on the causes, consequences and policy responses to health worker emigration, from which a key emergent theme was on the influence of gender on these three foci.

Data were collected in three phases. First, scoping reviews of the published literature and policy documents for the decade 2005 to 2015 were used to assess what was known/not known before we proceeded with collecting empirical data (*as detailed elsewhere, the scoping review applied an Arksey and O’Malley* [4] *approach to the search of Medline, PubMed and Embase databases using key terms “migration”, “health professional(s)”, “health worker(s)”, “health personnel” and terms for the three countries*). Second, we conducted 117 in-depth interviews with health worker stakeholders, senior policy officers and health-care systems specialists [42 India; 38 Philippines and 37 South Africa]. Third, we conducted on-line and household surveys with a total of 3580 health workers [1719 in India, 1244 in South Africa, and 617 in the Philippines) to assess their intent to migrate and the factors within the health system and broader society affecting those decisions (see Table 1).

**Table 1:** Overview of empirical data from South Africa, India and the Philippines

	Health worker surveys		Stakeholder interviews
	Male n (%)	Female n (%)	
South Africa	703	541	37
Physicians	568 (80.8)	272 (50.3)	
Nurses	6 (0.9)	166 (30.7)	
Other	129 (18.3)	103 (19.0)	
India	581	1 138	42
Physicians	297 (51.1)	235 (20.7)	
Nurses	49 (8.4)	510 (44.8)	
Other	235 (40.5)	393 (34.5)	
The Philippines	150	467	38
Physicians	17 (11.3)	28 (6.0)	
Nurses	119 (79.3)	349 (74.7)	
Other	14 (9.4)	90 (19.3)	
Total	1 434	2 146	117
Physicians	882 (61.5)	535 (24.9)	
Nurses	174 (12.1)	1025 (47.8)	
Other	378 (26.4)	586 (27.3)	

### Gender-based analysis

The documentary data and stakeholder interviews were originally analysed thematically with a common coding scheme developed in partnership between Canadian principal investigators and the country-based teams. Using a subset of gender-related keywords, we analysed the documentary and interview data for any explicit comments or details that related to gender and gender differences in behaviour or experiences, but also that spoke to gender roles and relations [37]. We examined any differences that related to the influence of gender on individual health workers through a sex-based disaggregation of the survey data, and to its influence on the questions posed to stakeholders that were sensitive to the issues of gender roles, relations and experiences.

Health worker surveys included a binary^3^ demographic question for female/male which was used to categorize responses to content questions. In each country these questions included reasons for considering emigration both in terms of work and living conditions, the consequences of geographical or regional impacts in relation to health worker shortages and or adequacy, and policy responses to endemic health worker emigration. The data analysed here are largely descriptive, reporting frequencies of responses of the push and pull factors influencing male and female workers differently, as well as their views on impacts and possible policy responses. These analyses are complemented with the qualitative responses of workers from their interviews as well as those with stakeholders.

## Results

### South Africa

The literature and documentary data on health worker emigration from South Africa reveals a number of gender-based insights. Hull [36], for example, found that the career decisions of female nurses are influenced by “opportunities or constraints” created through their personal relationships and networks. In short, female nurses’ choices to migrate or to migrate and return are often fraught with moral decision-making related to family and community responsibilities [11, 23, 36]. The cultural capital or status that comes with economic security from migration also helps improve women’s status outside of South Africa [36].

Family and friends already located in the proposed destination country serve to ‘pull’ prospective migrants [71]. The growth of overseas nurse associations and other support networks facilitates nurse emigration from South Africa to the UK [74]. For example, Public Services International (PSI), a global federation of public sector trade unions, initiated a 16-country campaign in 2005 for ethical recruitment in international migration, and in the recruitment of female health workers specifically [74].

The findings from our survey of South African health workers’ views of the importance of key push and pull factors affecting the emigration intentions of health workers did not reveal significant differences when men and women were compared (Table 2). Lack of respect from government, workplace infrastructure such as facilities, equipment, and supplies, and their personal security in the workplace were considered important push factors by close to 90% of all respondents. Ratings of living condition push factors revealed between 94 and 96% of male and female health workers rated the level of corruption as well as their personal safety and that of their family as important. The future of their children in their home country was rated as less important, but 91.3% of men and 87.8% of women also considered their children’s future important factors when deciding to move to a different country. Caution must be exercised in comparing these findings, given the higher predominance of male physicians in our sample.

**Table 2:** Push factors affecting emigration of male and female health workers from South Africa

Perceived importance of…	Health workers from South Africa	
	Male n (%)^a^	Female n (%)^b^
Working conditions		
Poor infrastructure	636 (90.5)	474 (87.6)
Personal security at work	627 (89.2)	483 (89.3)
Lack of respect from government	618 (87.9)	470 (86.8)
Living conditions		
Level of corruption	673 (95.7)	512 (94.7)
Lack of personal/family safety	675 (96.0)	511 (94.5)
Poor future for children	642 (91.3)	475 (87.8)

^a^Percentage of all male respondents

^b^Percentage of all female respondents

The stakeholders we interviewed recognized gender as an important factor which suffused aspects of career choice and professional development, but how this impacted emigration intentions and decisions was more complex. First, gender discrimination was generally understood by a number of stakeholders as a generator of inequality in the workplace, with efforts reportedly being made to address it:

*Basically, the Department of Health in South Africa as well, as in the provinces, are committed to achievement of targets in terms of (the) Employment Equity Act. So, gender discrimination …we are not supporting that. We are trying by all means to ensure that now we are dealing with those discrepancies accordingly. (SA KI 12)*

Nevertheless, those we interviewed also recognized the gendered nature of health worker career choice. One South African stakeholder suggested that women were preferred health workers because of their innate personal characteristics: “*Women tend to be more level-headed and cool-headed you know, as opposed to some of the men. They do bring a fair amount of stability within the department*.” (SA KI 14) Fewer men involved in nursing was acknowledged, for example, but equality within the profession was assumed:

*For gender—there is equity in nursing—equal pay for equal work, no discrimination in salary because you are a woman or man; and you know nursing remains female dominated, but as a profession nursing is very strong. (SA KI 4).*

In the case of physicians, one stakeholder commented on the assumed suitability of women for certain positions as registrar doctors (i.e., those who train to become a specialist or sub-specialist):

*(In) the surgical specialities only one specialist can take three registrars. So in terms of females it is also a bit of a problem because there are certain specialities that females don’t feel comfortable with, like surgical, because they have to raise children. You know males are a bit different because I can be working every day in the night so it might not be a problem. So females, they battle with those kind of things, so they rather … they prefer to do the soft things. (SA KI 5)*

Despite some insight into the gender differences in the division of labour in the South African health-care system, the stakeholders we interviewed did not consider that gender might influence health workers intentions to emigrate. As one stated:

*No. there is no difference about gender. Really, I have never heard that the ladies wanted to do it more than the gentlemen or the men or the women. There’s no definite difference between that. Definitely not so … I’ve got single ladies interested in going, I’ve got family people, I’ve got single guys…there’s definitely no one group more than other. (SA KI 6)*

However, another stakeholder offered a different opinion: “*since nursing is populated by ladies, then mostly it is females. But way back I know that even males have migrated to other countries*.” (SA KI 13). Another argued that women were reportedly more interested in emigrating because of the stability that jobs elsewhere could offer them. *“So they are busy being recruited and the majority of them, you know, because I think also because they want stability because most of them are mothers and most of them are females*.” (SA KI 14).

Another interviewee pointed out the preference in destination countries for female health workers. For example, “*If it is the Middle East females go to the Middle East because the culture and the religious beliefs there accept females than males. But UK takes both…both females and males*.” (SA KI 15).

Regardless of some recognition of the importance of gender on migration push and pull factors, there was no mention of how policy responses to health worker emigration might need to take gender into consideration.

### India

Much of the literature and documentary data in India that has any bearing on gender and migration is associated with nursing. The rate of nurses emigrating from India is sizeable with estimates that range from 20% of all nurses [33] to as high as 50% who graduate from certain schools [43]. Johnson, Green and Maben [38], for example, noted that nurses’ reasons for emigrating were a complex of several push and pull factors *“… migration as a short term option to satisfy career objectives—increased knowledge, skills and economic rewards—that could result in long-term professional and social status gains 'back home' in India”* (p. 734–735). Young unmarried nurses i.e. recently trained and with a little experience, were reported as most likely to leave India. Destination countries included those in the OECD and the Middle East. According to OECD [56] data, nearly 56,000 Indian-trained nurses work in the United States, United Kingdom, Canada, and Australia, which is equal to about 3% of the number of registered nurses in India [89].

Tables 3 and 4 outline the views of the push and pull factors affecting the emigration intentions of health workers from two different regions in India—Punjab and Kerala—broken down by gender. The most important factor for both men (42.5%) and women respondents (41.6%) from Punjab was their dissatisfaction with the future for their children if they remained in India. Fewer health workers from Kerala (21.3% of men and 14.9% of women) shared the concern for the future of their children. Male health workers in Kerala were specifically dissatisfied with their current income (76.3%) but this was lower amongst female health worker respondents (42.5%). Female health workers responded similarly to males with regards to the costs of living (49.3% female compared to 51% male). Female health workers from Punjab were concerned about their current income (37.1%) and the costs of living (31.9%), but in this region, fewer male health workers shared these concerns with 20.1% of men dissatisfied with their income and 14.9% with the costs of living. Women in Punjab were more worried about their personal safety (39.1%) compared to both male health workers in Punjab (15.7%) and to female health workers in Kerala (14.4%).

**Table 3:** Push factors affecting emigration of male and female health workers from India (Punjab)

Dissatisfaction with current…	Health workers from India (Punjab)	
	Male n (%)^a^	Female n (%)^b^
Working conditions		
Poor income	27 (20.1)	92 (37.1)
Poor working conditions	22 (16.4)	32 (12.9)
Poor education opportunities	21 (15.7)	45 (18.1)
Living conditions		
Future of your children in India	57 (42.5)	103 (41.6)
Personal safety	21 (15.7)	97 (39.1)
High living costs	20 (14.9)	79 (31.9)

^a^Percentage of all male respondents

^b^Percentage of all female respondents

**Table 4:** Push factors affecting emigration of male and female health workers from India (Kerala)

Dissatisfaction with current…	Health workers from India (Kerala)	
	Male n (%)^a^	Female n (%)^b^
Working conditions		
Poor income	190 (76.3)	378 (42.5)
Poor working conditions	125 (28.0)	213 (24.0)
Poor education opportunities	124 (27.8)	190 (21.3)
Living conditions		
High living costs	228 (51.0)	439 (49.3)
Future of your children in India	95 (21.3)	133 (14.9)
Personal safety	76 (17.0)	128 (14.4)

^a^Percentage of all male respondents

^b^Percentage of all female respondents

The findings from the stakeholder interviews largely supported the findings in the literature, but provided more nuanced description with regard to the gender conditions and impacts on nurse emigration from India. In the literature, social network factors appear to have played a critical role in Indian nurse emigration, as diaspora family members in Western countries facilitate the migration and retention of migrants. There is also evidence that emigration can enhance single women’s prospective marriage [84, 86]. One informant added that foreign-earned income, which is greater than what could be earned at home, could enhance the prospect of marriage for women. “*One reason for Kerala girls migrating in large numbers is that in Kerala the bridegroom has to be paid a huge dowry and these girls need to collect these funds*.”(KI KI #1).

Many interview participants described how female nurses stated their intentions to emigrate but also to later return to India. These intentions may be based on the opportunities for citizenship that are available in the countries to which they are migrating, and the unemployment and underemployment of nurses in the domestic context. Countries such as Saudi Arabia and other Middle-Eastern countries nearby to India promise good wages and have become major destination countries for Indian health workers, but they do not offer permanent residence or citizenship to migrants [5]. Good wages allow putative migrants to consider temporary emigration, earning enough money to remit and help to ensure financing for their own eventual return and retirement in India. The literature (e.g., [62]) and our findings strongly suggested that Indian nurses’ emigration to the Gulf states was always intended to be temporary, and timed to effectively intersect with and enhance life cycle events such as marriage and child birth.

Female nurses’ intentions to emigrate and actual emigration can be explained in part by the construction and production of Indian nurses: female nurses in India are raised, prepared and educated in a culture that increasingly encourages their emigration, and it is a culture which also discourages the inclusion of men: *For nursing, female nurses are preferred. In a vacancy of 20 posts they only want 4 male nurses, basically for Psychiatry ward.* Furthermore, male nurses were reported as badly treated in the Indian health context, *“nobody wants them.” and, “None of the private colleges are appointing male nurses*."

This also reflects informants’ comments that nurses, in general, have low status and receive little respect in relation to their work.

### The Philippines

Female health workers, and nurses in particular, have come to dominate migration flows from the Philippines [13, 49]. Therefore, gendered health workforce migration is typically equated with female nurse migration, with a majority (up to 75% depending on the year) of land-based migrants from the Philippines being educated women [7]. Le Espiritu [47], for example, notes that while female nurses from the Philippines mentioned economic motives, “…*many more cited desire to be liberated from gendered constraints: to see the world and experience untried ways of living*” (p. 8), including a “…*newfound freedom to make more independent choices about marriage*.” (p. 9).

Another factor that plays an important role in women’s decisions to migrate from the Philippines is related to their commitment to the sustainability and improvement of their family and community through the sending of remittances [8]. That is, while this motive for migration might seem purely economic, it is also rooted in cultural expectations and values related to gender roles. Indeed, Filipinas express pride in being responsible for their families back home, as Filipino culture places responsibility to care for their natal families on them. For example, culturally, it is prescribed that the eldest daughter should be providing for her parents and siblings [8].

In response to an unprecedented global demand for nurses, certain incentive schemes to retain health workers in the Philippines have been employed, some of which have included ‘gender sensitive considerations’ [34]. It has, for example, been recognized that since women represent a large proportion of health workers, the different needs of female health workers need to be considered and addressed when developing incentives to encourage workers to stay in the workforce. This includes flexible and part-time working options, flexible leave and vacation time, and access to childcare and schools [34].

Table 5 outlines the gender breakdown of the different push and pull factors considered by the health workers we surveyed in the Philippines, of which 78% were nurses. Similarly to some of the studies that emphasize the role of work and life related factors in emigration decision of health workers [21, 49], our survey respondents see these factors as being very important in such decisions. Overall, men and women rated these factors similarly. Most important was their satisfaction with their job for 72.1% of men and 74.4% of women. Half of both male and female respondents were dissatisfied with their current ability to find work in their chosen health profession in the Philippines. Regardless, few respondents considered it easy or very easy to find work in their health professional category overseas (19.7% of men and 17.2% of women). Men seemed to be slightly more dissatisfied with poor living conditions (41.1%) than women (33.9%).

**Table 5:** Push factors affecting emigration of male and female health workers from the Philippines

Push and pull factors of migration	Health workers from the Philippines	
	Male n (%)^a^	Female n (%)^b^
Working conditions		
Job satisfaction	49 (72.1)	128 (74.4)
Poor advancement opportunities	15 (22.4)	35 (20.7)
Easy to find health job overseas	13 (19.7)	29 (17.2)
Living conditions		
Lack of employment	36 (50.0)	93 (50.8)
Poor living conditions	30 (41.1)	61 (33.9)
High living costs	24 (32.9)	59 (32.4)

^a^Percentage of all male respondents

^b^Percentage of all female respondents

Our survey results suggest that social networks (family, friends and colleagues) may act as an important source of information for Filipino health workers who intend to emigrate. While our study did not explore a gendered aspect of such networks and their impact on the emigration decision of Filipino health workers, some research suggests that gender-based transnational social networks shape migration opportunities for female nurses as they help them to move and integrate into labour market of the destination country [47].

Policy stakeholders in the Philippines suggest that there are differing views on emigration as well as on the causes of the phenomenon, and these views are often related to gender. With regard to emigration-related policy, one official reiterated that the goal of bilateral agreements should always be fairness and social justice to all health workers and professionals, which included an awareness of concerns related to gender:

*There should be the principle of non-discrimination in employment terms and conditions; even in the issue of sex and gender, it should always be observed in the deployment of workers …* (P KI 08).

Given the active nature of the Philippines’ labour export policies, it is interesting that stakeholders rarely spoke specifically of gender, suggesting in part that sex and gender-based analysis of social contributors to the Philippines’ labour economy, and the impacts of women`s and men`s contributions in the informal sector have yet to be undertaken.

Despite the daily departure of over 75,000 Filipino migrant workers [78], stakeholders also did not often reflect on the normalization of migration, which some consider to be one of the key macro-level factors encouraging the emigration of health workers [21]. The Philippines’ government’s labour export policy that has been sustained over decades. This, in turn, is reinforced by the rhetoric that overseas Filipino workers are ‘heroes’ of the nation, and facilitated by a well-established state infrastructure that supports emigration. It is complemented by the seemingly unremitting demand for care-workers across the globe, which all together has contributed to the development of dominant discourse that strongly encourages Filipino health workers migration [69, 78], the majority of which are female nurses. Foreign labour markets are now seen as natural extensions of the domestic labour market [25]. Clearly, emigration from the Philippines is gendered as women assume financial responsibility, and as such are expected to emigrate and provide for the family from afar, while their female surrogates and in some cases, male family members stay home and take care of children [57, 59].

## Discussion

Our analysis of survey data from nurses, physicians and other health workers in South Africa, India and the Philippines and interview data with policy stakeholders reveals a curious absence of how gender influences health workers’ intentions to emigrate. This stands in contrast with its noted importance in the published literature on these three countries and some key policy documents. Perhaps it is more difficult for individual health workers to see the influence of gender on their personal decisions, and perhaps equally so for those working at the level of policy.

This absence may also be due to the influence of gender being masked by the impact of profession in our data. That is, although some push factors influencing health workers’ decision might carry more weight depending on their gender, it also intersects with the profession and their country's policy and cultural context. It is difficult, however, to tease apart the individual level gender differences that emerged from this examination from those related to the predominant gender of the profession—nursing being a gendered female profession and medicine, being gendered male.

For example, the fact that many health workers are women might explain inadequate remuneration levels and the generally low social status ascribed to the work of female health workers more generally [73]. In South Africa, where female health workers were described as “preferred” for “innate” personal characteristics and cultural reasons, and in India where males are directed away from these roles considered only for women, these trends may be linked to the general devaluing of this work, reflected in the pay inequity of nursing vis-à-vis other workers, such as physicians where men have traditionally predominated. Indeed, the negative employment conditions for nurses in India are directly liked to gendered hierarchies and patriarchal norms where nursing, a feminine occupation, is deemed subservient to medicine, a masculine occupation [88]. The identity of inadequate remuneration being a key driver of immigration may reflect the underlying gendered nature of the differences in remuneration levels across health worker cadres. The fact that these issues figured more prominently in India and the Philippines, where nurses predominated in our sample, more so than in South Africa where physicians predominated our sample, reveals the importance of examining not just the gender of the worker but the predominant gender of the profession in a more nuanced gender-based analyses.

Regardless of the transparency of gender differences and implications in migration decision-making and experiences for female and male health workers, gender has rarely been considered either as an important contextual influence or analytic category in policy responses. When it is considered, it is largely equated with the situation of women or ‘inherent’ feminine traits of health workers rather than a more balanced analysis that includes the influence of masculinity. This reflects a typical view of gender-based analyses focusing on the situation of women exclusively. Hiding in plain sight, making gender visible to health worker migration and associated health and social policies may be necessary. Raising awareness of both the surface and deeper level influences of gender is a preliminary step, but carrying out a systematic sex and gender-based analysis of health worker migration deserves attention and integration into policy responses. Policy-makers will need to receive evidence of the influence of gender on health worker migration, so that they can then understand that a gender-based analysis can be an important tool for health workforce planning and health system sustainability.

Increased international recognition and policies to implement gender-based analysis in health-related research sides with the arguments for explicit consideration of gendering of health workforce policies at all levels. Gender-based analysis is an approach that is now widely accepted and promoted by a wide range of bodies, including at the WHO [91] and spearheaded by the Gender Equity Hub of the Global Health Workforce Network. The Council of Europe (nd) has also adopted a ‘gender mainstreaming’ approach that calls for “the integration of a gender equality perspective into every stage of policy process…with a view to promoting equality between women and men” with all EU policies to take into account the different situations of women and men. In Canada, an intersectional gender-based analysis plus approach is written into the mandate letters of each federal minister [79] and in being mainstreamed throughout all departments.

Our findings, which initially prompted us to pursue analysing health worker migration from a gender lens, also presents a sufficient case for a review of theoretical approaches with respect to health worker migration and policy to include gender-based analysis. Assumptions about migration intentions and pathways tend to be premised on older migration theory and to a certain extent without a fulsome consideration of the influence of gender and its gendered implications across the whole migratory trajectory, from the household to the global context. This is a fruitful area for future consideration.

## Limitations and future research directions

Although it would have been instructive to be able to compare across countries and professions controlling for gender, the slight variations in survey questions adopted by the country-based teams and insufficient sample sizes made it difficult to more fully integrate the quantitative analyses. We were not, for example, able to tease apart whether the gender differences found in India were related to more male respondents being physicians and female respondents being nurses. Future research should attempt to advance an analysis that considers the gender of the professional, the gender of the profession, and the shifting gender dynamics of the professions, for example the feminisation of medicine and the masculinisation of nursing, and how this may relate to the opportunities presented by international migration.

## Conclusions

Our findings raise a number of questions and challenges including how to encourage and assist researchers and policy decision-makers, particularly those for whom gender is not a typical concern or those who do not possess skills in gender-based analysis, to think about and call attention to both what is known and what is not known and what is visible and not visible about gender in health worker migration. Policy-makers will need to receive evidence of the impacts of gender on skilled health worker migration, in order to understand that gender-based analysis is an important tool for health workforce planning and sustainability.

## Data Availability

The participating countries’ dataset(s) supporting the conclusions of this article are not publicly available to ensure respondents’ anonymity in reporting and confidentiality in participating in the study as per the study’s ethical requirements.

## Abbreviations

GBA: Gender-based analysis
KI KI: Kerala, India Key Informant
ILO: International Labour Organization
OECD: Organization for Economic Co-operation and Development
PAHO: Pan American Health Organization
P KI: Philippines Key Informant
PSI: Public Services International
SA KI: South African Key Informant

## Footnotes

1 We are fully aware that sex and gender are not dichotomous categories; however, they are treated as such (in this instance) in policies and documentation, etc.

2 As a side benefit of emigration, improvements in child health and infant mortality in some developing countries have been traced to the health education that migrant women obtain in destination countries (Association of Women’s Rights in Development, 2004, as cited in Jones et al. [39]).

3 We recognise that contemporary work on sex and gender-based analysis includes diverse sex and gender identities, orientations and expressions, but these concepts are more widely known in (Western) destination countries, informing a decision to focus on the binary identification for this study.

## References

[1] Adhikari R (2013). Empowered wives and frustrated husbands: nursing, gender and migrant Nepali in the UK. Int Migr.

[2] Akintola O (2004). A Gendered Analysis of the Burden of Care on Family and Volunteer Caregivers in Uganda and South Africa.

[3] Aiken LH, Buchan J, Sochalski J, Nichols B, Powell M (2004). Trends in International Nurse Migration. Health Aff.

[4] Arksey H, O'Malley L. Scoping studies: towards a methodological framework. Int J Soc Res Methodol. 2005;8(1):19–32.

[5] Babar ZR (2020). Understanding Labour Migration Policies in the Gulf Cooperation Council Countries. Asianization of Migrant Workers in the Gulf Countries.

[6] Bandita S. Women’s Labour Migration from Asia and the Pacific: Opportunities and Challenges (No. 12; IOM-MPI Issue in Brief). Migration Policy Institute (MPI) and the International Organization for Migration’s Regional Office for Asia and the Pacific. 2015. https://publications.iom.int/books/iom-mpi-issue-brief-no-12-womens-labour-migration-asia-and-pacific-opportunities-and

[7] Ball RE (2004). Divergent development, racialized rights: globalised labour markets and the trade of nurses—the case of the Philippines. Women’s Stud Int Forum.

[8] Basa C, Harcourt W, Zarro A (2011). Remittances and transnational families in Italy and the Philippines: Breaking the global care chain. Gend Dev.

[9] Bourgeault IL, Benoit C, Wrede S, Neiterman E, Dent M, Kuhlmann E, Denis JL, Bourgeault IL (2016). Migration of Expert Labour. The Routledge Handbook on Professions and Professionalism.

[10] Bourgeault I, Labonte R, Packer C, Runnels V, Tomblin Murphy G (2016). Knowledge and potential impact of the WHO Global Code of Practice on the International Recruitment of Health Personnel: Does it matter for source and destination country stakeholders?. Hum Resour Health.

[11] Brock G (2017). Just responses to problems associated with the brain drain: identity, community, and obligation in an unjust world. South Afr J Philos.

[12] Brugha R, Crowe S (2015). Relevance and effectiveness of the WHO global code practice on the international recruitment of health personnel—ethical and systems perspectives. Int J Health Policy Manag.

[13] Brush BL, Sochalski J (2007). International nurse migration. Policy Polit Nurs Pract.

[14] Buchan J. International recruitment of nurses: United Kingdom case study. Research Report No. 00814. London: RCN; 2002.

[15] Byron M, Chamberlain M (1998). Migration, work and gender: the case of post-war labour migration from the Caribbean to Britain. Globalised identities.

[16] Camlin CS, Snow RC, Hosegood V (2014). Gendered patterns of migration in rural South Africa. Popul Space Place.

[17] Castro-Palaganas EC, Spitzer DL, Kabamalan MMM, Sanchez MC, Caricativo R, Runnels V, Labonté R, Murphy GT, Bourgeault IL (2017). An examination of the causes, consequences, and policy responses to the migration of highly trained health personnel from the Philippines: the high cost of living/leaving—a mixed method study. Hum Resour Health.

[18] Cooper CL, Swanson N (nd) Workplace violence in the health sector state of the art. https://www.who.int/violence_injury_prevention/injury/en/WVstateart.pdf

[19] Council of Europe (nd) What is gender mainstreaming?. https://www.coe.int/en/web/genderequality/what-is-gender-mainstreaming#:~:text=Gender%20mainstreaming%20means%20integrating%20a,of%20policies%2C%20programmes%20and%20projects.&text=It%20is%20therefore%20a%20tool%20for%20achieving%20gender%20equality.

[20] Davda LS, Gallagher JE, Radford DR (2018). Migration motives and integration of international human resources of health in the United Kingdom: systematic review and meta-synthesis of qualitative studies using framework analysis. Hum Resour Health.

[21] Dimaya RM, McEwen MK, Curry LA, Bradley EH (2012). Managing health worker migration: a qualitative study of the Philippine response to nurse brain drain. Hum Resour Health.

[22] Donato KM, Gabaccia D, Holdaway J, Manalansan M, Pessar PR (2006). A glass half full? Gender in migration studies. Int Migr Rev.

[23] Dunne N. Who cares? Indian nurses ‘on the move’ and how their transnational migration for care work shapes their multigenerational relationships of familial care over time. Unpublished PhD thesis, Sociology, University of Edinburgh; 2019.

[24] Eckenwiler L (2011). Women on the move: Long-term care, migrant women, and global justice. Int J Feminist Approaches Bioethics.

[25] Engman M. A Tale of Three Markets: How Government Policy Creates Winners and Losers in Philippine Health Sector (Working paper). Retrieved from in Philippine Health Sector (Working paper). http://www.gem.sciences-po.fr/content/publications/pdf/Engman_Philippine_Nurses102010.pdf

[26] Ferrant G, Tuccio M, Loiseau E, Nowacka K. The role of discriminatory social institutions in female South-South migration. OECD Development Centre. 2014. http://www.oecd.org/dev/development-gender/SIGI%20and%20Female%20Migration_final.pdf

[27] Folbre N (2012). Should women care less? intrinsic motivation and gender inequality. Br J Indus Relations.

[28] George A. Human resources for health: a gender analysis. 2007. http://www.who.int/social_determinants/resources/human_resources_for_health_wgkn_2007.pdf

[29] George S (2005). When Women Come First: Gender and Class in Transnational Migration.

[30] Hagan JM (1998). Social networks, gender, and immigrant incorporation: resources and constraints. Am Sociol Rev.

[31] Hagopian A, Ofosu A, Fatusi A, Biritwum R, Essel A, Hart LG, Watts C (2005). The Flight of Physicians from West Africa: Views of African Physicians and Implications for Policy. Soc Sci Med.

[32] Hankivsky O (2013). The lexicon of mainstreaming equality: Gender Based Analysis (GBA), Gender and Diversity Analysis (GDA) and Intersectionality Based Analysis (IBA). Canadian Political Science Review.

[33] Hawkes M, Kolenko M, Shockness M, Diwaker K (2009). Nursing brain drain from India. Hum Resour Health.

[34] Henderson L, Tulloch J (2008). Incentives for retaining and motivating health workers in Pacific and Asian countries. Hum Resour Health.

[35] Hochschild AR, Hutton W, Giddens A (2000). Global Care Chains and Emotional Surplus Value. On The Edge: Living with Global Capitalism.

[36] Hull E (2010). International migration, "domestic struggles" and status aspiration among nurses in South Africa. J Southern Afr Stud.

[37] Johnson JL, Greaves L, Repta R (2009). Better science with sex and gender: facilitating the use of a sex and gender-based analysis in health research. Int J Equity Health.

[38] Johnson SE, Green J, Maben J (2014). A Suitable job? A qualitative study of becoming a nurse in the context of a globalizing profession in India. Int J Nurs Stud.

[39] Jones AD, Bifulco A, Gabe J (2009). Caribbean nurses migrating to the UK: a gender-focused literature review. Int Nurs Rev.

[40] Hennebry J, Walton-Roberts M, Newbold B, Wilson K (2019). Rebalancing act: promoting an international research agenda on women migrant care workers’ health and rights. A Research Agenda for Migration and Health.

[41] Kingma M (2006). Nurses on the Move: Migration and the Global Health Care Economy.

[42] Kofman E (2004). Gendered global migrations: diversity and stratification. Int Feminist J Politics.

[43] Kruppa K, Madhivanan P (2009). Leveraging Human Capital to Reduce Maternal Mortality in India: Enhanced public health system or public-private partnerships?. Hum Resour Health.

[44] Kuhlmann E, Bourgeault IL, Larsen C, Schofield T. Gendering health human resource policy and management. In: The Palgrave handbook of gender and healthcare. London: Palgrave Macmillan. 2012. p. 72–91.

[45] Labonté R, Packer C, Klassen N (2006). Managing Health Professionals Migration from sub-Saharan Africa to Canada: A Stakeholder Inquiry into Policy Options. Hum Resour Health.

[46] Labonté R, Sanders D, Mathole T, Crush J, Chikanda A, Dambisya Y, Runnels V, Packer C, McKenzie A, Tomblin Murphy G, Bourgeault IL (2015). Health worker migration from South Africa: causes, consequences and policy responses. Hum Resour Health.

[47] Le Espiritu Y (2005). Gender, migration, and work. Revue Eur Migrat Int.

[48] Lee S (2017). The three worlds of emigration policy: towards a theory of sending state regimes. J Ethnic Migration Stud.

[49] Lorenzo FM, Galvez-Tan J, Icamina K, Javier L (2007). Nurse migration from a source country perspective: philippine country case study. Health Serv Res.

[50] Lu-Farrer G, Yeoh BS, Baas M (2020). Social construction of skill: an analytical approach toward the question of skill in cross-border labour mobilities. J Ethnic Migration Stud.

[51] Makina A (2009). Caring for people with HIV: State policies and their dependence on women’s unpaid work. Gend Dev.

[52] McAuliffe M, Ruhs M, McAuliffe M, Ruhs M (2018). Migration and migrants: An overview. World Migration Report 2018.

[53] Nair S (2012). Moving with the times: gender, status and migration of nurses in India.

[54] Needham I, Kingma M, O’Brien-Pallas L, McKenna K, Tucker R, Oud N. Workplace violence in the health sector: together, creating a safe work environment. In: Proceedings: International Conference on Workplace Violence in the Health Sector, Amsterdam. 2008. http://www.oudconsultancy.nl/Resources/Proceedings_Workplace_Violence_2008.pdf

[55] Nelson JL, Rhodes R, Battin MP, Silvers A (2002). Just expectations: Family caregivers, practical identities, and social justice in the provision of health care. Medicine and Social Justice: Essays on the Distribution of Health Care.

[56] OECD. International Migration Outlook 2017. Paris: OECD Publishing. 2017. Available from: 10.1787/migr_outlook-2017-en.

[57] Oishi N. Gender and Migration: An Integrative Approach. (The Center for Comparative Immigration Studies CCIS Working Paper No. 49). 2002. https://ccis.ucsd.edu/_files/wp49.pdf

[58] PAHO (2001). Report on technical meeting of managed migration of skilled nursing personnel.

[59] Parreñas RS (2005). Children of Global Migration: Transnational Families and Gendered Woes.

[60] Paton B (1994). Labour export policy in the development of Southern Africa.

[61] Percot M (2006). Indian nurses in the gulf: two generations of female migration. South Asia Res.

[62] Percot M, Rajan SI (2007). Female emigration from India: case study of nurses. Econ Political Weekly.

[63] Pillinger J. Quality health care and workers on the move: South Africa National Report. Public Services International International Migration and Women Health and Social Care Workers Programme. 2011. http://www.world-psi.org/sites/default/files/documents/research/en_migrationhealthsocial_southafricareport_sept2011.pdf

[64] Pillinger J. Quality Health Care and Workers on the Move: Australia National Report. Public Services International International Migration and Women Health and Social Care Workers Programme. 2012. https://www.world-psi.org/sites/default/files/documents/research/ghana.pdf

[65] Piper N. Gender and migration: A paper prepared for the policy analysis and research programme of the global commission on international migration. Geneva: Global Commission on International Migration, 12. 2005. https://www.iom.int/jahia/webdav/site/myjahiasite/shared/shared/mainsite/policy_and_research/gcim/tp/TP10.pdf

[66] Pratt G, Barnes T (2004). From registered nurse to registered nanny: discursive geographies of Filipina domestic workers in Vancouver, B.C. Reading Economic Geography.

[67] Reverby S (1987). A caring dilemma: womanhood and nursing in historical perspective. Nurs Res.

[68] Robinson V, Carey M (2000). Skilled International Migration: Indian Doctors in the UK. Int Migr.

[69] Rodriguez M (2010). Migrants for Export: How the Philippines State Brokers Labor to the World.

[70] Ryan L (2008). 'I had a sister in England': family-led migration, social networks and nurses. J Ethnic Migration Stud.

[71] Sanders D, Lloyd B. South African health review 2005. In: Ijumba P, Barron P, eds. Human resources: International context. Durban (South Africa): Health Systems Trust. pp. 76–87. 2005. http://www.hst.org.za/publications/682.

[72] Schiebinger L (2014). Scientific research must take gender into account. Nature News.

[73] Shannon G, Minckas N, Tan D, Haghparast-Bidgoli H, Batura N, Mannell J (2019). Feminisation of the health workforce and wage conditions of health professions: an exploratory analysis. Hum Resour Health.

[74] Singh G. Health worker migration in South and Southern Africa. IOM; 2007.

[75] Spitzer DL, Çalışkan G (2020). Precarious lives, fertile resistance: migrant domestic workers, gender, citizenship, and well-being. Globalizing Gender, Gendering Globalization.

[76] Spitzer DL, Gideon J (2016). Engendered movements: migration, gender, and health in a globalized world. Handbook of Gender and Health.

[77] Spitzer DL, Thomas F, Gideon J (2016). Migration and health through an intersectional lens. Handbook of Migration and Health.

[78] Spitzer DL, Piper N (2014). Retrenched and returned: Filipino migrant workers during times of crisis. Sociology.

[79] Status of Women Canada. What is GBA+? 2020. https://cfc-swc.gc.ca/gba-acs/index-en.html

[80] Thompson R, McConnell ES, Corazzini KN (2017). Factors Shaping Caribbean Nurse Migration and the Impact On Long-Term Care. Innovat Aging.

[81] Timur S (2000). Changing Trends and Major Issues in International Migration: An Overview of the UNESCO Programmes. Int Soc Sci J.

[82] Tomblin Murphy G, MacKenzie A, Waysome B, Guy-Walker J, Palmer R, Elliott Rose A, Rigby J, Labonté R, Bourgeault IL (2016). A mixed-methods study of health worker migration from Jamaica. Hum Resour Health.

[83] United Nations. The Universal Declaration of Human Rights. 1948. https://www.ohchr.org/EN/UDHR/Documents/UDHR_Translations/eng.pdf

[84] Walton-Roberts M (2012). Contextualizing the global nursing care chain: international migration and the status of nursing in Kerala India. Global Netw.

[85] Walton-Roberts M (2015). International migration of health professionals and the marketization and privatization of health education in India: from push-pull to global political economy. Soc Sci Med.

[86] Walton-Roberts M (2015). Femininity, mobility and family fears: Indian international student migration and transnational parental control. J Cult Geogr.

[87] Walton-Roberts M (2019). Asymmetrical therapeutic mobilities: masculine advantage in nurse migration from India. Mobilities.

[88] Walton-Roberts M (2019). Occupational (im)mobility in the global care economy. J Ethnic Migr Stud..

[89] Walton-Roberts M, Rajan SI. Global Demand for Medical Professionals Drives Indians Abroad Despite Acute Domestic Health-Care Worker Shortages. Migration Information Source. 2020. https://www.migrationpolicy.org/article/global-demand-medical-professionals-drives-Indians-abroad

[90] Walton-Roberts M, Runnels V, Rajan SI, Sood A, Nair S, Thomas P, Packer C, MacKenzie A, Murphy GT, Labonté R, Bourgeault IL (2017). Causes, consequences, and policy responses to the migration of health workers: key findings from India. Hum Resour Health.

[91] World Health Organization (2019). Women Deliver, Men lead: a gender and equity analysis of the global health and social workforce. Hum Resour Health Observer Series.

[92] World Health Organization. The WHO Global Code of Practice on the International Recruitment of Health Personnel (Sixty-third World Health Assembly-WHA63.16; p. 15). World Health Organization. 2010. http://www.who.int/hrh/migration/code/code_en.pdf

[93] World Health Organization (2006). World Health Report: Working Together for Health.

[94] Yeates N (2004). Broadening the scope of global care chain analysis: nurse migration in the Irish context. Fem Rev.

[95] Yeates N (2009). Globalizing care economies and migrant workers: explorations in global care chains.

[96] Yeoh BSA, Battisella G (2014). Engendering international migration: Perspectives from within Asia. Global and Asian Perspectives on Migration.

